# “Like a lots happened with my whole childhood”: violence, trauma, and addiction in pregnant and postpartum women from Vancouver’s Downtown Eastside

**DOI:** 10.1186/1477-7517-12-1

**Published:** 2015-01-12

**Authors:** Iris Torchalla, Isabelle Aube Linden, Verena Strehlau, Erika K Neilson, Michael Krausz

**Affiliations:** Centre for Health Evaluation and Outcome Sciences (CHÉOS), St. Paul’s Hospital, 588-1081 Burrard Street, Vancouver, BC V6Z 1Y6 Canada; Department of Psychiatry, University of British Columbia, Detwiller Pavilion, 2255 Westbrook Mall, Vancouver, BC V6T 2A1 Canada; School of Population and Public Health, University of British Columbia, James Mather Building; 5804 Fairview Avenue, Vancouver, BC V6T 1Z3 Canada

**Keywords:** Substance use, Women, Pregnancy, Trauma, Gender-based violence

## Abstract

**Background:**

Women living in poor and vulnerable neighbourhoods like Vancouver’s Downtown Eastside (DTES) face multiple burdens related to the social determinants of health. Many of them struggle with addiction, are involved in the sex trade and experience homelessness and gender-based violence. Such evidence suggests that psychological trauma is also a common experience for these women.

**Methods:**

The purpose of this qualitative study was to explore themes and subjective perspectives of trauma and gender-based violence in women who lived in an impoverished neighbourhood and struggled with substance use during pregnancy and early motherhood. We interviewed 27 individuals accessing harm reduction services for pregnant and postpartum women in Vancouver, Canada.

**Results:**

Key themes that emerged from these women’s narratives highlighted the ubiquity of multiple and continuing forms of adversities and trauma from childhood to adulthood, in a variety of contexts, through a variety of offenders and on multiple levels. Both individual and environmental/structural conditions mutually intensified each other, interfering with a natural resolution of trauma-related symptoms and substance use. Women were also concerned that trauma could be passed on from one generation to the next, yet expressed hesitation when asked about their interest in trauma-specific counselling.

**Conclusions:**

In offering harm reduction services for poor and marginalized women, it is clear that an understanding of trauma must be integrated. It is recommended that service providers integrate trauma-informed care into their programme in order to offer this service in a trusted environment. However, it is also necessary to shift the focus from the individual to include environmental, social, economic and policy interventions on multiple levels and from issues of drug use and reduction of drug-related harms to include issues of gendered vulnerabilities and human rights.

## Background

Extreme poverty neighbourhoods have been defined by poverty rates of at least 40% [1]. Such neighbourhoods have received increasing attention by researchers and policy makers because concentration of poverty is associated with additional social problems that are in turn linked to negative health outcomes for their residents [2]. The Downtown Eastside (DTES) of Vancouver, British Columbia, is a small geographical area of about 17,000 people [3] that has often been described as the poorest neighbourhood in Canada [4]. Many community members experience unemployment, precarious housing, violence and crime. A plethora of readily available, low-cost, illicit drugs also exist there, resulting in high rates of crack cocaine, crystal methamphetamine and heroin use [5], and the mortality rates for drug-induced deaths are seven times greater than the provincial rates [6]. Involvement in sex work and the drug trade are common means for the DTES residents to make ends meet, and many struggle with mental illness, medical diseases such as HIV/AIDS and hepatitis and substance use disorders (SUDs) [6]. Researchers have also identified the significance of traumatic experiences such as childhood maltreatment, sexual assault and family and partner violence for individuals with substance use problems [7, 8]. Such evidence suggests that people residing in the DTES face multiple burdens related to social determinants of health and that psychological trauma is also likely a common experience.

About 38% of the DTES population are women [3]. In a community health survey of DTES residents, 78% of the women reported recent use of illicit drugs and about one third reported injection drug use [9]. Female injection drug users in Vancouver have mortality rates almost 50 times that of the province’s general female population [10]. Researchers have pointed out how the numerical superiority of men in the DTES has created a dominantly male street culture and gendered risk environments in which women experience particular marginalization, exploitation, increased safety risks [11] and gender-based violence [8, 12]. Gender-based violence against women has been defined as any act “that results in, or is likely to result in, physical, sexual or psychological harm or suffering to women, including threats of such acts, coercion or arbitrary deprivation of liberty, whether occurring in public or in private life” [13]. In practice, it includes an array of offenses against girls and women such as sexual and physical violence, psychological abuse, violence in pregnancy, coerced sex/rape, sexual slavery, sexual harassment and forced prostitution conducted by family members, intimate partners and other perpetrators [14]. Women who use substances generally tend to report more sexual victimization and multitype trauma [15, 16] and greater rates of posttraumatic stress disorder (PTSD) [17] than men. In a study of homeless people with SUDs from British Columbia, PTSD comorbidity was significantly more prevalent in women than men; PTSD and gender were both associated with variables of psychopathology, and the most severe pattern was found for women with PTSD [18]. Women from the DTES also have high rates of pregnancy and poor pregnancy outcomes [19, 20]. Studies among mothers living in poverty found that histories of abuse and maltreatment were associated with greater psychological distress and drug use severity [21], low parenting satisfaction and physical punishment and neglect of their own children [22, 23].

It must be pointed out that reports on the deficits and problems in the DTES represent only one side of the coin and do not reflect the strength, resilience and courage of many individuals living in this neighbourhood. The DTES has an energetic and creative community of advocates and activists that initiate and support change. For example, an organization was formed in 1997 by Vancouver drug users and activists to address the health crisis among intravenous drug users in the DTES by organizing public demonstrations and discussions, operating various educational and support programmes, advocating for changes in drug policies and participating in council and policy planning meetings [24]. Other work has focused on developing affordable housing, promoting harm reduction services and creating safe spaces for women. In 2000, the City of Vancouver released its Four Pillars Drug Strategy (prevention, treatment, harm reduction and enforcement), a pragmatic approach to drug issues to reduce the health, social and economic costs of legal and illegal substance use without necessarily reducing the consumption [25]. Vancouver’s current harm reduction programmes include a supervised injection site, needle exchanges and low-threshold community housing and health services. For example, pregnancy outreach programmes with a harm reduction philosophy have been established to respond to a growing understanding of the complex health and social needs of pregnant women and new mothers who use substances. The overall goals of these programmes are to improve pregnancy outcomes, increase the women’s well-being and help them reduce their risk behaviours, support mothers in their capacity as parents and promote the health and development of their children. However, despite existing evidence highlighting the importance of trauma in the lives of women with SUD, the majority of research projects and programme planning in the DTES has focused on injection drug use and infectious diseases whereas mental health issues and trauma have often been neglected [26]. The goal of the current paper is to complement the existing work by exploring themes of trauma, violence and mental health in pregnant and postpartum women who struggled with substance use and accessed harm reduction services. Staff who are working with these women might not fully understand the associated challenges or have the training to address the complexity of problems among individuals with substance use issues [27]. Understanding their perspectives can offer insight into the complexities of providing services for marginalized individuals; feeding this information back to the harm reduction services could help to improve service access and intervention outcomes for these women.

## Methods

The study was conducted in Vancouver between June 2009 and March 2010. Eligible participants were adult women who were struggling with substances during pregnancy and/or early motherhood. They had to be 19 years of age or older and have given birth in the past 5 years. Participants were recruited from three community programmes which provided harm reduction services for substance-using mothers: 1) ‘Sheway’ is a drop-in centre in the DTES that offers prenatal and postnatal care, sexual health counselling, addiction counselling and methadone maintenance treatment, practical support, food and nutrition counselling, parenting classes, and First Nation specific services for about 120–160 women. 2) ‘Fir Square’ is a residential programme at a Women’s Hospital dedicated to providing care for substance-using women and their newborns in a single unit. The programme helps women and their newborns stabilize and withdraw from substances, while keeping mothers and babies together and continuing to provide care from antepartum to postpartum and between hospital and community. 3) ‘Crabtree Corner Housing’ is located in the DTES and provides transitional housing for pregnant and parenting women who use substances. The centre also offers meal programmes, child care, support groups and programmes related to parenting, family activities, health prevention and child development.

Study recruitment posters were placed at these services. Recruitment was slow, partly due to the slow relationship building the researchers experienced with the services. The research team also presented the study design and recruitment strategy to the staff of Sheway because it is the primary service provider for substance using mothers in Vancouver. From this meeting, it was suggested that the interviewers become more familiar with the study population by engaging in some of the activities during Sheway’s drop-in hours. In the initial recruitment period, the interviewers helped with child minding, organizing and distributing donations, food and other supplies, as well as accompanying some mothers and children to the park. The interviewers also set up a display booth during high volume drop in hours, presenting information about the study and screening interested mothers. Furthermore, at the end of each study interview, participants were given cards detailing the contact information of the study coordinator and asked to share that information with any other women they thought might be eligible to participate. The original target number for the study was 50 women, but during data analysis, it became clear that after interviewing 27 women, no new knowledge was being generated and therefore data saturation was reached.

After providing written informed consent, participants completed several quantitative questionnaires and a semi-structured qualitative interview. All interviewers had previous experience working with marginalized populations and had performed similar interviews before. Interviews were conducted and audiotaped in private rooms at Sheway, Fir Square or in participants’ homes by three female research assistants. Child care was offered when the interviews were conducted at the services. All participants received an honorarium of $20.

A total of 33 women met the inclusion criteria and consented to participate in the study. Only 31 participants completed the quantitative questionnaires because two women were unable to attend their scheduled appointment and could not be reached to re-schedule. The quantitative measures assessed sociodemographic characteristics and information related to substance use, childhood maltreatment, adult abuse experiences and general and PTSD-related distress. The results have been reported elsewhere [28].

Qualitative methodology was employed within this population specifically because of the sensitive nature of this topic and the lack of available in-depth information. This article describes the qualitative component of the study aimed at giving a voice to the women, obtaining their personal and subjective experiences and perspectives on trauma and addiction and appreciating individual differences in addition to collecting standardized data. The qualitative interviews were guided by pre-identified themes (i.e. the context of substance use, the context of trauma and violence and the interplay of trauma and substance use) which prompted the following questions: *Can you describe to me how your drug use started? Can you tell me how your drug use developed when you learned you were pregnant? How was your mental health, how did you feel? Did you experience any significant life event that may have affected you?* The interviewer also asked questions about the women’s relationships with their family members and partners, explored their interest in trauma treatment and encouraged women to discuss other issues that were important to them but have not been addressed by the interviewers. Interviews took between 1 and 4 h. The interviewers determined in repeated discussion with the principal investigator (M.K.) that data saturation was reached after interviewing 27 women.

These women were on average 32.0 years old and about half of them self-identified as aboriginal. Nineteen women had a foster care history, more than half of them had no high school diploma, and 24 received their current income from governmental support. The women were up to 1 year postpartum and 21 women had more than one child. Sixteen women reported that they had no partner at the time of the interview (see Table 1 for detailed sociodemographic information).

**Table 1:** **Sociodemographic information of the study sample (** ***n*** **= 27)**

	M	SD
Age	32.0	5.7
Maternal age	22.4	5.3
	*N*	%
Ethnicity^a^		
White	13	48.1
First Nations/aboriginal	13	48.1
Asian	3	11.1
Marital status		
Single	14	51.9
Partnered	7	25.9
Married	4	14.8
Divorced	2	7.4
Education		
No high school diploma	15	55.6
High school diploma	2	7.4
Some college education	3	11.1
College graduation	4	14.8
Graduate studies	1	3.7
Other	2	7.4
Total number of children		
1	6	22.2
2	4	14.8
3	10	37.0
4	3	11.1
5–8	4	14.8

^a^The numbers add up to 29 because two women declared two ethnicities.

All interviews were transcribed verbatim and entered into NVIVO. The goal of the analysis was to provide a thematic description of key elements involved in our participant’s lifetime experiences. The interviews were first examined for content through multiple readings and thematic analysis. Themes were identified by two raters (I.T. and I.L.), a clinical psychologist and researcher with a Master of Public Health, by extracting central topics that were recurring within and across the individual interviews. Initial coding was conducted independently, with codes being applied incident by incident. After an initial coding phase, a codebook was created, incidents were pooled and redundant codes were collapsed. The findings are presented narratively. The study had received approval from the University of British Columbia and Providence Health Care ethics boards.

## Results

Six key themes were found; the themes are as follows: (1) women spoke of adverse and traumatic experiences in early childhood, (2) the continuation of adversities and trauma in adulthood, (3) intimate partner violence, (4) structural violence, (5) transgenerational trauma and (6) their interest in trauma counselling. The results illustrate the complexities of the target population, all of which are important considerations when offering harm reduction services.

### Adversities and trauma during childhood

The vast majority of women described difficult circumstances and multiple problems in their families when they were growing up. Often, one or both of the parents or primary caregivers used substances themselves and/or sold drugs to make a living, and they were exposed or introduced to drugs by their families and peer groups at an early age. The majority of participants reported that their drug use started in very early adolescence. Some participants reported that their parents had mental illnesses. Some participants mentioned that they have had mental health problems themselves from very early on. Exposure to domestic violence, sexual, physical and emotional abuse and neglect was common. All of the women reported childhood maltreatment experiences; more than three quarter (*n* = 24; 77.4%) reported sexual abuse and each of the other types of childhood maltreatment (i.e. physical and emotional abuse and physical and emotional neglect) was reported by at least 17 of the women (see [28] for detailed results of the Childhood Trauma Questionnaire). In most cases, the participants had experienced multiple forms of adversities and trauma.

“*I was heavily abused as a child. Mentally and physically by my mother. And, sexually by her boyfriends. And I watched her getting raped. And I had, I watched her get beat the crap out of her, I walked in on her slitting her wrists. And the blood was just, spraying everywhere. And, uh, yeah, it, I had, uh, a traumatic childhood*” (White woman, 36 years).

These experiences led some participants to drift into subcultures and peer groups where exposure to family dysfunction, abuse and neglect was the norm. Participants also ran away from their parents, were evicted by their parents and/or were placed in foster homes. This absence of a stable home resulted in further disruption of normal developmental processes, making participants more likely to engage in deviant behaviours such as drug use, criminal and violent behaviours and prostitution and exposed them to further risk.

“*My grandparents raised me part, on a small reserve. And I was in foster care since I was 13. I moved myself from the reserve. Then my father called and asked for a visitation. At the time I was trying to get out of the foster system. Having to be in somebody else’s care, it was a lot of rules. I’d never had rules growing up. So I ran away. I moved here and I ended up in the Main and Hastings area*” (Aboriginal, 30).

“*I was like a full, crack head. I put myself in foster care. And uh then, I’d run away, and stay out for 30 days, at a time until, the um, kiddie cop car, would pick me up and, take me back to my foster parents’ house. Or my group home. Um, and, uh, like I was prostituting on the streets at that point too. Uh, starting at 12*” (White, 33).

### Continuing adversities and trauma in adulthood

Normality and daily routines were missing in the lives of most participants, and many of them were unable to complete school, get an education and take up employment. Their childhood was often chaotic and characterized by abuse and neglect and it continued to be so in adulthood. Twenty-seven women (87.1%) indicated they had experienced emotional abuse, 23 (74.2%) reported physical abuse and 16 (51.6%) reported sexual abuse in adulthood, oftentimes by multiple perpetrators. Environmental and structural factors contributed to the dangers and threats that dominated the women’s everyday life. For example, all of the women reported experiences of homelessness or precarious housing at some point in their lives; many of them early in their career, multiple times and/or for long periods of time. Life on the streets in combination with drug use often involved engagement in risky activities (e.g. prostitution) and exposure to high risk and traumatic situations (e.g. sexual and physical assault) which typically deteriorated their condition.

“*I had ended up in a building in Vancouver that was one of the worst places you could rent from, full of hookers and drug addicts. And it was a nightmare. I was selling myself on the street. I had no income at all whatsoever. There was no beds for me to go to. There was no detoxes at the time. I was suicidal and I just wanted to die*” (White, 37).

“*I ended up back in escort. And I was dating somebody and he showed me how to deal. But I ended up using coke and heroin again. I started drinking. I was making mad cash back then. I’d work and work and work, seven days a week, and I’d stay up for three days and I’d sit there and get high. And my kids would be, in the other room, and it was, just crazy. Dope was always in front of me now*” (Aboriginal, 31).

Most women were victims of violence, trauma and continuing and multiple adversities from very early on and, as a consequence, never had a regular and ‘normal’ life. However, a minority of women indicated that they had completed their education and obtained a job, but lost everything after experiencing trauma and/or addiction.

“*I have been abducted by a psychopath. He had me for seven months, and he was giving me, GHB, PCP and 25 other pharmaceuticals. I went from being a supervisor in a mental health facility to, a homeless crack head. Lost my apartment, lost everything I owned*” (White, 36).

“*I had a stable job. And I was a university graduate. And then I met my boyfriend and we moved out. And I found out he was an alcoholic and a crack user. He would get violent, and I was so bruised that I couldn’t go to work, I couldn’t speak. He locked me up and stole all my bank cards and fed me crack cocaine and got me addicted. For seven days and nights straight the first time, I didn’t know my name, I didn’t know anything, and then …yeah, I lost my job because of this man, and I lost all my life savings, and I lost my home*” (Southeast Asian, 32).

### Intimate partner violence

Women’s narratives of their relationship with intimate partners reflected gendered relations of power between the women and their intimate partners. Male partners, rather than female friends, were mentioned as significant sources of influence throughout their lives. Some women were introduced to drugs by their boyfriends. Oftentimes, their partners used substances as well and were involved in criminal activities. Although several women perceived their partners as supportive—emotionally as well as in their daily struggle and their efforts to reduce their substance use or take care of the children—the majority experienced exploitation and repeated physical, sexual and/or emotional abuse by their partners. Pregnancy was a time of hope for many women. Several participants indicated that they were hoping for a new beginning and turning point in their lives and their relationship. They expected that parenting a baby will help them and their partners to abstain from substances, stop their partners’ violence and bring them someone who would love them. But in most cases, their hopes for a better life were not fulfilled. The women often did not receive much support by the fathers of their children and carried all the responsibilities themselves. Their partners did not stop the abuse, the women and the babies continued to be exposed to violence and harm and the trauma continued.

“*We moved to Vancouver because we wanted to, raise this baby and, get away from the drugs and alcohol. This is where we came to start fresh and I thought maybe he would be better. And then, he was getting worse, physical, verbal abuse, you know, emotional, everything. When the baby was two and a half months, I just had enough, because I kept ending up with black eyes and a fat lip. And I was just tired of being, abused and my daughter seeing that*” (Aboriginal, 29).

“*After six months pregnant I put him in jail, because I was beaten so bad I was hospitalized. I had big bruises on the side of my stomach. So I had to keep getting checked, cause if he damaged the baby’s head in any way, there could be brain damage*” (Aboriginal, 30).

Raising children in the context of drug use, violence, homelessness and prostitution led to most babies being apprehended from their mother’s care; some women had several children in the care of foster families or relatives. The experience of their children being taken away from them was upsetting for all women, and they felt it was unfair and wrong.

“*Losing him, was my biggest, downfall. Cause I was clean when I lost him. And, to lose a child after raising him for two years has absolutely destroyed me*” (Aboriginal, 37).

“*I always, talked to her in my belly and said, we’re gonna, mommy’s gonna really gonna do good and mommy’s gonna love you. And, keep you and, you know. So, that was one hard thing when um, when yeah, when she was taken away from me*” (Aboriginal, 29).

### Structural violence

Some women reported experiences of stigmatization and gender-based psychological violence from the health care system. This type of experience reinforced the treatment that the women had made from their personal relationships and on the streets.

“*I did have a family doctor for some time, until I told her I was escorting for work. And she totally turned her nose up at me. I wanted to get a pap smear and a blood test, but she basically sent me out. So I went somewhere else and got it done. My safety and my wellbeing is still, huge. I might be a drug addict, but, like, regardless I want to make sure that I’m healthy and get checked. Because, you know, it’s a dangerous lifestyle. And you would expect your doctor to help you*” (White, 37).

One woman was brought to the hospital after being raped during her pregnancy. The police were called and arrived at the hospital to question her about the rape. “*When I came in, they didn’t want to check me right away. I was bleeding, I was in a lot of pain, I was crying, but they just said ‘No, the officers are here.’ And the cops kept harassing me, saying ‘Oh, were you using drugs? You deserve…’ well not to get raped, but they were basically saying ‘oh, you asked for it.’ And all I kept saying was that I wanted to get checked for the baby, and they kept saying, ‘You have to wait, the officers want to talk to you’ and I kept saying ‘Check my baby, I’ll talk to them later’, right? I was really upset – I just got raped, and they’re trying to question me?*” (White, 36).

In contrast, many women explicitly stated that they experienced no stigmatization at Sheway, Fir Square and Crabtree Corner.

“*But even when I checked myself in [at Fir Square], the nurse that intook me to, was really good. Cause I started crying - and she said, I’m glad you’re here. The staff, at Fir is, so amazing! Like no, judgment at all*” (White/Aboriginal, 31).

### Transgenerational trauma

Reflecting on their experiences, several women observed patterns between their parents and themselves or themselves and their own children—trauma being passed on from one generation to another—and several mentioned how important it is for them to “break the cycle”.

“*I look healthy. I’m normal. But I don’t know. Sometimes I feel a little wacked up here. You know and that could just be, my upbringing right? It was really violent so I can have violent tendencies. So from childhood to pre-teen and then pre-adult and then adult it’s just been an ongoing cycle that I haven’t broke*” (Aboriginal, 32).

“*Even though you’ve been living on the streets, or you know, come from a very unhealthy background, with a very unhealthy childhood like I did, it is possible to turn things around and break that cycle*” (White, 20).

### Interest in trauma counselling

The vast majority of women have never been offered any treatment that specifically addressed the trauma that they had experienced. When being asked about their interest in receiving trauma counselling, the majority expressed ambivalence, reluctance or refusal; many answered with a simple “No”. Few women stated that they wished to see or currently saw a trauma counsellor.

“*I haven’t done trauma counseling. I should. I don’t think I’m, ready right now. Like a lots happened with my whole childhood*” (Aboriginal, 32).

“*I was very traumatized. I still am, right now. I was afraid, I would never sleep, I had insomnia – I would stay up and hold a bat by my door, cuz I was scared. And now that I’ve had trauma counselling, I’m hoping that I am able to recover*” (White, 35)*.*

## Discussion

This study explored themes of trauma, violence and mental health in pregnant and postpartum women who struggled with substance use and accessed harm reduction services, in order to offer insight into the complexities of the target group and elucidate potential points for improvement of and access to services. The key themes that emerged from the women’s narratives highlighted the ubiquity of multiple and continuing forms of adversities and trauma, often in form of gender-based violence, in a variety of contexts, from a variety of offenders and on multiple levels. The definition of gender-based violence against women is based on an understanding that such violence is influenced by gender roles and discrepancies in power and status and supports the legitimization and perpetuation of gender inequalities [14]. Gender-based violence is not only a serious human rights problem but has also been identified as a public health issue that is associated with a variety of adverse psychological, physical and social consequences [29].

The majority of our study participants talked about experiencing multiple adverse events and conditions in childhood, and all of the women indicated having experienced one or more forms of childhood abuse and neglect on the Childhood Trauma Questionnaire [28]. For many years, studies on childhood adversities have focused on one or two specific types of maltreatment, typically sexual and/or physical abuse. However, research has shown that sexual and physical abuse often co-occur with neglect, emotional maltreatment and other childhood adversities [30–32]. The self-medication model is often used to explain how individuals who have experienced trauma use substances to regulate the resulting distress [33]. The self-medication model appears to reflect accurately the experiences of our own study participants, but their stories also suggest that the risky behaviours and conditions that are associated with illicit drug use (e.g. intoxication, prostitution, homelessness, etc.) increased the risk for revictimization—as reflected by the “high-risk hypothesis” [34], and once the relationship between both factors was established, they reinforced and maintained each other.

The self-medication model and similar models [35, 36] developed to explain the long-term sequelae of childhood adversities focus on individual factors. Our results suggest that environmental and structural factors contribute to the harm participants experienced as well, beyond individual factors. Rhodes [37] conceptualized the ‘risk environment’ as comprising different types of environments—physical, social, economic and policy—which interact on both the micro- and the macro-level to produce drug-related harm. Micro-environmental parameters include factors such as locations of drug use and sex work, social and peer-group risk norms, income generation, access to social housing, etc. Macro-environmental parameters include gender inequalities and gendered risks, stigmatization of drug users, public health policy governing harm reduction and drug treatment, etc. The risk environment framework shifts the responsibility for drug-related harms from the individual to include the structures in which they have developed, and it extends the focus of harm-reduction activities from the individual to include sociopolitical and structural changes [37].

Women who have experienced childhood adversities have a greater risk of being a victim of intimate partner violence in adulthood [38], and many of our study participants had experienced abusive relationships and violence by their intimate partners. Researchers have highlighted how violence, including intimate partner violence, was considered a “normal” experience in the everyday life of inner city women who use drugs [39]. Epele extended the focus from the individual level to include social factors on the micro-environmental level by discussing how a male-centred street ideology places women in a subordinate position, pointing out that in her study, only a few drug-using women defined their relationship with their male partner as equal sharing. Instead, the relationship provided them with protection from street-based violence at the risk of experiencing domestic violence [40]. In a field observation in the San Francisco street drug scene, the authors described how women sought older male partners to protect them from violence and sexual harassment whereas men sought young women as sexual, romantic and income-generating partners, and how these relationships were usually abusive and exploitative to the women [41]. Given this, gender-focused violence prevention programmes in the city may benefit from components on the micro-level that address the social networks of substance-using women. One possibility may be to incorporate the involvement of peers to allow the exploration of unequal gender relations and peer norms. Such a model has for instance been successfully employed by Oxfam with the general female population in Africa and South America through women-specific social services [42].

Our results also suggest that structural (e.g. economic and policy) factors contribute to the dangers and threats that dominate the women’s everyday life. Many of our participants have lived on the street or in precarious conditions; some of them even became homeless as teenagers. Homelessness during adolescence may disrupt the youth’s developmental processes and expose them to further harm once homeless [43]. Researchers have proposed a risk amplification model where intrafamilial childhood maltreatment places adolescents at risk for becoming homeless, and subsequent events and behaviours on the street exacerbate the effects of adverse childhood experiences and resulting psychological distress and increase the risk of revictimization and retraumatization [44]. Homelessness and marginalized housing continued to be problematic in adulthood for our participants, and many of them experienced violence on the street. In their qualitative study, Lazarus and colleagues discuss how—within the gendered risk environments of the male-centred housing models—low-income and transitional housing resulted in marginalization, sexual and economic exploitation and increased safety risks for women [11] which is consistent with our participants’ accounts. Many of our own study participants were engaged in street-based sex work for some period of their lives, a dangerous business that involves a high risk of victimization, especially for women who are homeless [12]. In a synthesis of the literature of women in the street drug economy, Maher and Hudson pointed out how women remained marginalized with respect to opportunities for income generation, being confined to the harsh economy of street-based sex work [45]. Such findings on structural factors promoting violence against women highlight the need for structural (e.g. economic and policy-based) interventions on the micro- and the macro-level. These could include creating safe and innovative housing models for women, implementation of victim support services for sex workers and homeless women and reducing economic inequities to reduce the need for working in the sex trade. Furthermore, our participant’s reports indicate that structural violence is reproduced through interactions in the health care system, suggesting the need for training programmes for all professionals working with women who use substances.

Women also described the distress that they experienced of their children being apprehended by the child welfare system. Perspectives on the issue were that the process was unfair and wrong. The apprehension of children may compound an overlaying of trauma in adulthood for these women. Aligned with this thought, in 2003, an article discussed children being taken from Vancouver drug-using mothers and stated that Canadian drug users are afflicted by the government’s neo-liberal repressive drug policy combined with a patronizing and paternalistic social and child welfare system [46], which suggests that policy reforms on the macro-level may be necessary to improve the situation of drug-using mothers and their children. Furthermore, innovative models of care are needed on the micro-level which aim at increasing the women’s well-being and resilience, supporting them in their capacity as caregivers, improving their economic and social position and ultimately helping them to retain custody of their children.

In summary, our findings suggest that drug use and trauma follow complex patterns among women seeking harm reduction services in the DTES, where women have experienced multiple and often severe early childhood adversities in the form of both single traumatic events and chronic stressors; they experienced distress resulting from these adversities and used substances to self-medicate their distress. Once regular substance use was established, they entered a vicious cycle of engaging in high-risk behaviours and situations to secure drug supply, resulting in more trauma exposure and a lifestyle that was characterized by gendered risks, ongoing adversities and violence. All of these conditions mutually intensified and maintained each other and interfered with natural, healthy resolution of trauma/PTSD symptoms and substance use. Our study provides an exploration of the gendered nature of violence and trauma and the need to create the environmental conditions for reducing health inequalities on multiple levels.

In our study, women spoke of concerns of passing on trauma from one generation to the next, which confirms a desire to address their trauma, and yet women also articulated hesitancy towards seeking trauma specific support. In offering harm reduction services, it is clear that an understanding of trauma must be integrated. It is recommended to health care providers and policy makers that women with concurrent substance use and trauma issues receive comprehensive and integrated treatment that addresses both conditions within the same service in order to better meet the needs of this population. This study contributes to a growing body of literature that can be found in support of this recommendation [47, 48]. In recent years, a number of integrated treatment programmes for trauma and addiction have been developed and tested and yielded some promising results, although the majority of these programmes involve treatment components to promote stabilization and safety rather than trauma processing [49]. Our participants’ hesitation or refusal when asked about their interest in receiving trauma counselling raises questions about how their experiences can be best addressed. It is possible that trauma-specific interventions would not be well utilized. Researchers and clinicians have advocated for adopting a comprehensive trauma-informed approach in all harm reduction facilities providing services for women [50]. Trauma-informed interventions are distinct from trauma-specific interventions such that the care delivery practices take into account an understanding of the impact of trauma on an individual’s life, development and substance use but do not necessarily require disclosure of trauma. In contrast, trauma-specific interventions address trauma experiences directly and facilitate trauma recovery through counselling and treatment [50, 51]. Our participant’s responses suggest that trauma-informed approaches may be more appropriate than trauma-specific interventions in low-threshold harm reduction services for women in the DTES. They can be offered even to clients who choose not to work on their trauma issues immediately and may also pave the way for considering additional steps towards recovery from trauma and proceeding to trauma-specific treatment.

A few important limitations should be considered in evaluating the findings of the study. All qualitative studies are limited in terms of making generalizations about the entire population from a small, non-random sample of respondents. The self-selected sampling procedures likely excluded the voices of many women in the DTES, such as women who do not seek out any services or those living in other residential neighbourhoods. The experiences of women who do not have children and those who are younger than 19 years were also not represented. Furthermore, the study was conducted in a very specific setting: harm reduction services in a progressive city at the West Coast of Canada.

## Conclusions

In summary, interventions for women who use substances need to account for the complex needs of their clients. In recognition of the continuing impact that experiences of trauma, violence and abuse have on poor and underserved women in Vancouver’s DTES, the recommendation is made that presently trusted and successful service providers could integrate trauma-informed care into their programme in order to offer this crucial service in a trusted environment. Trauma-specific treatment should be offered to women who are ready to engage in this intervention. However, it is also necessary to shift the focus from the individual to include environmental, social, economic and policy factors on multiple levels and from issues of drug use and reduction of drug-related harms to include issues of gendered vulnerabilities and human rights.

## Abbreviations

DTES: downtown Eastside
PTSD: posttraumatic stress disorder
SUD: substance use disorder.
